# Iron Deficiency Anemia in Infants—Diagnostic Challenge and Assessment of Dietary Impact on Its Prevalence

**DOI:** 10.3390/healthcare14050574

**Published:** 2026-02-25

**Authors:** Kinga Ilnicka-Borowczyk, Małgorzata Dobrzyńska, Katarzyna A. Kaczmarek-Kryszak, Anna Szczepańska-Álvarez, Tomasz Podgórski, Jagoda Osipa, Hanna Markowska, Dagmara Woźniak, Sławomira Drzymała-Czyż

**Affiliations:** 1 Department of Bromatology, Poznan University of Medical Sciences, Marcelińska 42, 60-354 Poznan, Poland; kingailnickaborowczyk@gmail.com (K.I.-B.); mdobrzynska@ump.edu.pl (M.D.); katarzynakaczmarek.official@gmail.com (K.A.K.-K.); osipajagoda@gmail.com (J.O.); hmarkowska8@gmail.com (H.M.); drzymala@ump.edu.pl (S.D.-C.); 2 Complex of Healthcare Institutions, Kościuszki 96, 64-700 Czarnków, Poland; 3 Department of Mathematical and Statistical Methods, Poznan University of Life Sciences, 60-637 Poznan, Poland; anna.szczepanska-alvarez@up.poznan.pl; 4 Department of Biochemistry, Poznan University of Physical Education, 61-871 Poznan, Poland; podgorski@awf.poznan.pl

**Keywords:** nutritional programming, transferrin, ferritin, hepcidin, child nutrition, development, educational intervention

## Abstract

**Background/Objectives**: Iron deficiency affects 2% of infants under six months of age and 4–18% of infants aged 6–12 months and may lead to anemia. Given the consequences of iron deficiency in infancy and the importance of adequate nutrition, this study aimed to assess indicators of iron metabolism in infants whose parents participated in nutritional education. **Methods**: The study included 104 infants, divided into a study group (SG, *n* = 52) receiving a nutritional education intervention and a control group (CG, *n* = 52). Peripheral blood morphology parameters and biochemical markers, e.g., iron status (serum iron, transferrin, ferritin, and hepcidin), were evaluated at 3 and 12 months of age. Additionally, at study end, parents completed a three-day dietary diary to assess their infant’s iron intake. **Results**: After nearly one year of intervention, no cases of anemia based on hemoglobin concentration were identified in either group. However, infants in the SG were less likely to present iron and ferritin concentrations below reference ranges compared to the CG. In the CG, low ferritin levels occurred more frequently at 12 months than at baseline. This finding may be related to higher dietary iron intake in the SG, as insufficient iron intake was more common among the CG. Heatmap analysis revealed strong positive correlations among erythrocyte indices, confirming their internal consistency. No single parameter emerged as a superior marker of iron deficiency, emphasizing the need for a combined assessment of iron status. **Conclusions**: Parental nutritional education may improve iron status and reduce the risk of iron metabolism disorders in infants.

## 1. Introduction

Iron is an essential micronutrient required by all living organisms. In the human body, it is present in hemoglobin, myoglobin, tissue enzymes, and stored in the form of ferritin [1]. Although iron is traditionally associated primarily with cellular respiration, it also plays a critical role in DNA synthesis, antimicrobial defense, cholesterol metabolism, and the detoxification of harmful substances in the liver [2,3].

Moreover, iron is indispensable for proper brain development. Iron deficiency may disrupt neurotransmitter homeostasis, reduce myelin production, impair synaptogenesis, and weaken basal ganglia function [4,5,6]. Anemia resulting from iron deficiency has a detrimental impact on cognitive abilities and psychomotor development. Research has demonstrated a high prevalence of iron deficiency among individuals with attention-deficit/hyperactivity disorder (ADHD) and autism spectrum disorders [7,8]. Furthermore, iron deficiency can lead to or exacerbate deficiencies in other essential nutrients, adversely affecting not only the developing brain but also other organs [9,10]. This issue is particularly significant during infancy and early childhood.

Iron deficiency is one of the most widespread nutritional deficiencies globally, affecting an estimated 2 billion people according to the World Health Organization [11,12]. Most epidemiological studies on iron deficiency focus on women of reproductive age [13,14,15,16]; however, data on infants and young children remain limited. Existing evidence suggests that iron deficiency occurs in approximately 2% of infants under six months of age [17] and between 4% and 18% in infants aged 6 to 12 months [18]. Data for children aged 1 to 3 years vary significantly, with prevalence estimates ranging from 4% to 41% [18].

Manifest iron deficiency anemia, particularly microcytic anemia, represents a serious consequence of insufficient iron intake, yet it tends to develop at a relatively late stage [19,20]. Therefore, it is essential to identify iron deficiency promptly and effectively in infants and young children and simultaneously ensure adequate dietary iron intake. Iron requirements are substantially elevated in early childhood, while dietary sources of iron are often insufficient [21].

In light of the potentially severe consequences of iron deficiency in infancy, its diagnostic challenges, and the potentially beneficial effects of appropriate nutrition, this study aimed to assess the concentrations of various indicators of iron metabolism in infants whose parents had undergone long-term nutritional education.

The objective of the study was accomplished through the assessment of peripheral blood morphology parameters, as well as the concentrations of iron, transferrin, ferritin, total iron-binding capacity (TIBC), and unsaturated iron-binding capacity (UIBC) in two groups of infants: those who underwent a nutritional intervention and those who served as the control group. These measurements were performed twice when the infants were 3 and 12 months of age. Importantly, the results are presented in terms of both their clinical and critical significance—namely, the frequency of values falling outside the reference range, which indicates the need for therapeutic intervention. Additionally, the analysis identifies which of the assessed parameters correlate most strongly with iron deficiency.

## 2. Materials and Methods

### 2.1. Study Population

Although 128 full-term infants up to 12 weeks of age (over 95% of whom were enrolled at 6 weeks of age) were initially enrolled in the study, only 115 were ultimately randomized. Two infants met exclusion criteria—birth weight under 2500 g and the presence of severe chronic gastrointestinal diseases affecting digestion and absorption—and 11 infants failed to meet inclusion criteria, including the requirement for parental or legal guardian consent.

All enrolled infants were recruited from family medicine clinics in the Greater Poland Voivodeship between 2023 and 2024. Recruitment typically took place during routine vaccination visits (K.I.-B., H.M.).

The study was conducted in accordance with the principles of medical research design [22].

### 2.2. Intervention Description

The study design has been described in detail previously [23]. In brief, after randomization using a 1:1 allocation ratio via a randomization table from randomizer.org, infants were assigned to one of two groups. Parents in the intervention group installed a mobile application through which brief messages on infant nutrition guidelines were sent 4–6 times per week. These messages were tailored to the infant’s age, season of the year, and mode of feeding (breastfeeding versus formula feeding). Examples of such messages included simple prompts such as: “Iron absorption from the diet is inhibited when iron-rich foods are consumed together with calcium-rich products such as milk and dairy foods” or “Excess simple sugars—glucose, fructose, and sucrose—found in juices, fruits, and sweets can impair the absorption of nutrients such as iron and zinc, suppress appetite, and promote obesity.” Emphasis was placed on preventing iron deficiency and so parents received information on iron-rich foods, encouragement to intensively expand the infant’s diet, and guidance on vitamin C sources to enhance iron absorption.

Control-group parents received only a general infant feeding schematic.

The application used in the project was developed by a software engineer from the Faculty of Computer Science and Telecommunications at Poznań University of Technology. The application included an interface that allowed tracking of notification reading. If three consecutive notifications were not opened, parents automatically received an email reminder encouraging them to resume engagement with the educational content. Patient adherence to the educational program was continuously monitored. The readability of the nutritional messages among parents in the intervention group was high, with an average rate of 88% ± 25%. Additionally, some participants used optional supplementary support, including contact with a dietitian via chat, text messaging, or telephone consultation.

After approximately 10 months of nutritional education, the intervention’s efficacy was assessed by comparing selected iron-metabolism parameters in the two groups.

### 2.3. Blood Sampling

Blood samples were collected twice from each participant: at enrollment (up to 12 weeks of age) and at study conclusion (in 12 months of age). Two tubes were drawn on each occasion—one EDTA tube and one serum tube—each with a maximum volume of 2.7 mL (Sarstedt, Nümbrecht, Germany) to minimize discomfort. The EDTA blood sample was immediately analyzed for hematological measurement, while the clotted sample was centrifuged under standard conditions and the serum was frozen until biochemical assays. All phlebotomy was performed by qualified nursing staff.

### 2.4. Biochemical and Hematological Measurements

Complete blood counts were obtained using a 20-parameter automated hematology analyzer (Mythic^®^ 18, Orphée, Geneva, Switzerland). Key indices included hemoglobin concentration (HGB), mean corpuscular volume (MCV), and hematocrit (HCT).

Serum iron concentration was measured colorimetrically (ACCENT-200 FERRUM, Cormay, Łomianki, Poland; sensitivity test: 4.1 μg∙dL^−1^). Transferrin (ACCENT-200 TRANSFERRIN, Cormay; sensitivity test: 0.076 g∙L^−1^), ferritin (ACCENT-200 FERRITIN, Cormay; sensitivity test: 9.1 ng∙mL^−1^), and unsaturated iron-binding capacity (UIBC; ACCENT-200 UIBC, Cormay; sensitivity test: 20 μg∙mL^−1^) were determined by specific antigen–antibody and colorimetric reactions. All biochemical parameters were quantified on an Accent 220S analyzer (Cormay) as previously described [24]. Total iron-binding capacity (TIBC) was calculated by summing measured serum iron and unsaturated iron-binding capacity (UIBC).

Hepcidin (Shanghai SunRedBio, Shanghai, China; sensitivity test: 5.123 ng∙mL^−1^), ferroportin (Shanghai SunRedBio; sensitivity test: 0.175 ng∙mL^−1^) and C-reactive protein (hsCRP, DBC Inc., New York, NY, USA, sensitivity test: 10 ng∙mL^−1^) concentrations were determined using commercially available ELISA kits. Spectrophotometric measurements with ELISA tests were made using a multi-mode microplate reader (Synergy 2, SIAFRT; BioTek, Winooski, VT, USA).

Iron deficiency and anemia were diagnosed according to recommendations of the Polish Pediatric Society, the Polish Society of Pediatric Oncology and Hematology, the Polish Society of Neonatology, and the Polish Society for Family Medicine [25], and based on new reference intervals established with the Dade Dimension Clinical Chemistry System [26].

### 2.5. Dietary Diary Analysis

Nutritional intake was evaluated using a three-day food diary completed by the parents for their infants, following the guidelines developed by the National Institute of Food and Nutrition [27]. The diary included detailed information on the types of food products consumed, portion sizes (grams or milliliters), and the exact times of consumption. To minimize error, a physician (K.I.-B., H.M., J.O.) and a dietitian (D.W., M.D.) instructed parents on accurate diary completion. The instructions emphasized comprehensive recording of all meals, snacks, fluids, and milk, and encouraged parents to use online tools (e.g., ilewazy.pl) to ensure precise estimation of portion sizes. Parents could consult a registered dietitian by phone if needed.

Submitted diaries were analyzed by dietitians using Dietetyk 2015 software (Jumar Software, Poznań, Poland). Mean daily macro- and micronutrient intakes were compared to Polish Recommended Dietary Allowances. Nutrients directly related to iron metabolism or absorption—such as protein, fiber, and vitamin C—were selected for further analysis.

### 2.6. Sample Size Calculation

The primary outcome was the measurement of hemoglobin concentration. For the sample size calculation, it was assumed that the expected difference between the groups in hemoglobin levels would be 7%, with a standard deviation of 10%. These assumptions were based on preliminary results from a study using a similar method of hemoglobin assessment. Minimal sample size was determined with G*Power 3 (University of Düsseldorf, Düsseldorf, Germany). Assuming 80% power, a two-sided α of 0.05, and anticipating a 20% dropout rate, data from 104 infants were required, increasing the enrollment target to 124 participants.

### 2.7. Ethical Considerations

The study was conducted in accordance with the Declaration of Helsinki and received approval from the Poznań University of Medical Sciences Ethics Committee (Approval No. 394/22).

### 2.8. Statistical Analysis

For all parameters, median (interquartile range), mean, and standard deviation were reported. Normality was assessed with the Shapiro–Wilk test. Between-group differences were evaluated using the Mann–Whitney U test or Kruskal–Wallis test, as appropriate. A *p*-value < 0.05 was considered statistically significant. Analyses were performed using GraphPad Prism 5.01 (GraphPad Software, La Jolla, CA, USA).

The correlation analysis was performed according to the intention-to-treat (ITT) principle; missing data were supplemented using the Missing Completely at Random (MCAR) method. The applied algorithm for data imputation was missRanger (2021). The correlation matrix was calculated via Spearman’s rho. All calculations were performed using Jamovi version 2.7.13.0.

In the groups under consideration, a two-proportion test was used to compare the probability of observing values of the analyzed parameter below the reference range. The alternative hypothesis assumed that the probability in the first group was lower than in the second group. When the data suggested the opposite relationship, a test with the reverse assumption in the alternative hypothesis was applied. The analysis included results in which the number of observations below the reference range satisfied the condition:

$$m\geq \frac{5}{min\;(\pi ,\;1-\pi )}$$

where *m* is the number of observations below the reference limit, and *π* is the probability of obtaining an observation below the reference limit [28].

Additionally, the relationship between parameters was examined in the subgroup of patients whose blood iron levels were below the reference range. Spearman’s rank correlation coefficient was calculated. The results were visualized using heatmaps, highlighting similarities between the parameters. All analyses were performed in R.

## 3. Results

A total of 104 infants (52 vs. 52) completed the study. The study group (SG) consisted of 25 girls and 27 boys, whereas the control group (CG) included 27 girls and 25 boys. The body weight of children who completed the study at the time of enrollment did not differ significantly between groups (study group vs. control group: ns), both when expressed in grams and as body weight *Z*-scores (Table 1). Through collaboration with primary care physicians from large urban centers (>500,000 inhabitants), smaller towns and suburban areas, we recruited parents from diverse settings. The distribution of place of residence was similar between groups (SG: 15%, 24%, 61% vs. CG: 15%, 28%, 57%; *p* = ns). Educational attainment in our cohort also reflects the Polish population of young parents, with a predominance of higher education (SG 73% vs. CG 76%; *p* = ns).

The proportion of mothers reporting breastfeeding immediately after birth was very high in both groups (SG 94% vs. CG 93%). At study enrollment (typically 6–8 weeks of age) this proportion had declined but remained comparable between groups (78% vs. 76%).

The analysis of the probability of observing values below the lower reference limit in infants at 12 months of age (follow-up) showed that the likelihood of iron and ferritin concentrations falling below the reference range was lower in the study group compared to the control group (Table 2). Furthermore, in the control group, ferritin concentrations below the reference range occurred less frequently at baseline than at 12 months of age (follow-up). Using assumptions contrary to the alternative hypothesis, it was also demonstrated that the probability of iron concentrations falling below the lower limit in the study group was higher at baseline than at follow-up. Notably, no differences were observed at baseline in the probability of values of the analyzed parameters falling below the lower reference limit between the study and control groups. Furthermore, it was shown that the concentrations of all analyzed parameters at baseline did not differ significantly between the groups (baseline: study group vs. control group—ns). At 12 months of age, after nearly one year of dietary intervention, the study group exhibited significantly higher levels [23] of hemoglobin (follow-up: study group vs. control group, (*p* = 0.0499) and ferritin (*p* = 0.0067), as well as lower levels of TIBC (*p* = 0.0478) and ferroportin (*p* = 0.0410). Additionally, in both the study and control groups, ferritin levels decreased over the course of the study (study group: baseline vs. follow-up, *p* < 0.0001; control group: baseline vs. follow-up, *p* < 0.0001), whereas transferrin, UIBC, TIBC, hepcidin, and ferroportin levels increased significantly.

In the subsequent stage of the analysis, only infants diagnosed with anemia—defined as iron concentrations below the reference range—were included, regardless of group allocation. Lower iron concentrations correlated only with higher CRP values. No associations were observed for the remaining hematological parameters or other indicators of iron status (Table 3).

**Figure 1:**
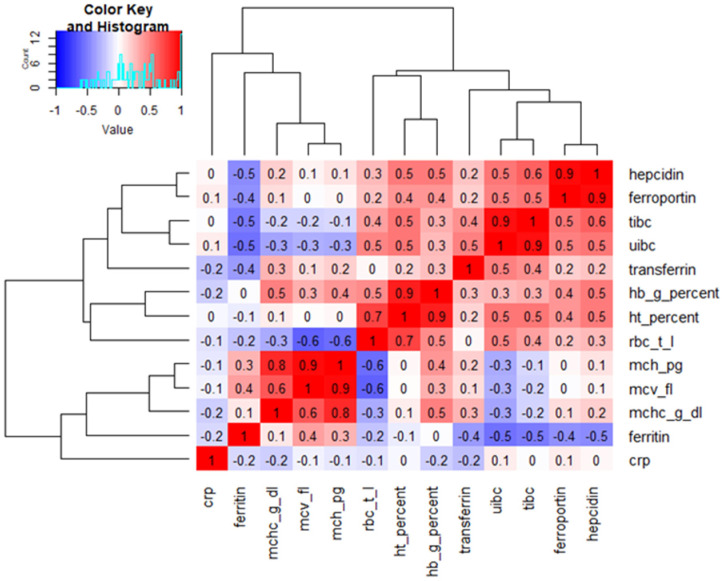
Heatmap of correlations between all analysed parameters in infants at 12 months who exhibited iron concentrations below the lower reference limit. tibc—total iron binding capacity; uibc—unsaturated iron-binding capacity; hb—hemoglobin; ht—hematocrit; rbc—red blood cells; mch—mean corpuscular hemoglobin; mcv—mean cell volume; mchc—mean cell hemoglobin concentration; crp—high-sensitivity C-reactive protein.

Heatmap analysis was performed on the dataset of infants from the study and control groups at 12 months of age (follow-up) who exhibited iron concentrations below the lower reference limit (Figure 1). The heatmap allowed visualization of correlations between all analyzed parameters. This analysis revealed a very strong positive correlation between hepcidin and ferroportin, UIBC and TIBC, and hematocrit and hemoglobin, as well as MCH and MCV (r = 0.9 for all). A rather strong negative correlation was also demonstrated between RBC and MCH and MCV (r = −0.6 for both).

**Figure 2:**
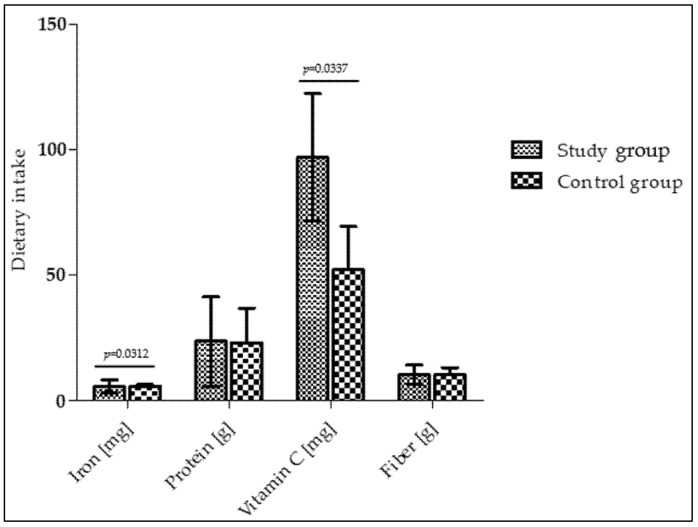
Dietary intakes in infants at 12 months of age.

Analysis of the children’s food diaries at 12 months of age (follow-up) showed that infants in the study group consumed significantly more iron and vitamin C than the control group of infants (median [1st–3rd quartile]: 7.58 [3.62–10.10] mg vs. 5.70 [5.26–6.2] mg and 100.1 [76.2–149.2] mg vs. 57.9 [38.8–97.5] mg, respectively (Figure 2)). However, protein and fiber intake remained comparable between the study and control groups (median [1st–3rd quartile]: 42.2 [12.9–45.1] g vs. 36.4 [13.7–43.2] g and 11.7 [7.3–18.5] g vs. 12.1 [7.8–19.5] g, respectively). After intensive nutritional education, the prevalence of insufficient iron intake—defined as consumption below 80% of the recommended dietary allowance (RDA)—was significantly lower in the study group compared with the control group (23% vs. 58%, respectively; *p* = 0.028).

## 4. Discussion

Our previous findings indicated that parental nutritional education resulted in higher hemoglobin and ferritin concentrations in 12-month-old infants, along with lower levels of parameters associated with increased iron absorption, namely TIBC and ferroportin [23]. In the present study, the aim was to examine whether the implemented dietary intervention has a clinical impact and whether it can prevent iron deficiency and, consequently, anemia. Although the demonstration of higher hemoglobin concentrations in the intervention group compared with the control group is an important research finding, this difference is not necessarily clinically significant, especially when the hemoglobin concentrations in all infants examined in both groups were within the reference range. Therefore, the analysis focused on determining how many infants had iron status and biochemical parameters below the reference range, rather than comparing their measured values.

The current analysis demonstrated that dietary intervention (in the study group) was associated with a decreased likelihood of iron and ferritin concentrations dropping below established reference values. Moreover, despite the physiological reduction in body iron reserves during the first year of life, the likelihood of iron concentrations falling below the lower reference value was higher in the first quarter of life than at 12 months of age among infants in the intervention group. This observation may be explained by the increased iron intake among infants receiving the intervention, which is further supported by the finding that insufficient iron intake was more common in the control group.

As mentioned in the introduction, the consequences of iron deficiency can be serious and may significantly affect infant development. An attempt was made to determine which of the analyzed parameters best reflect iron deficiency. Statistical analyses revealed only a significant inverse correlation between lower iron concentration and hsCRP levels.

Correlation analysis visualized by a heatmap with hierarchical clustering revealed distinct clusters of hematological and iron metabolism parameters. Strong positive correlations were observed among erythrocyte indices (hemoglobin, hematocrit, MCV, MCH, and MCHC), confirming their internal consistency. Parameters related to iron transport (TIBC, UIBC, and transferrin) formed a separate cluster and were inversely correlated with ferritin, reflecting reduced iron stores. Hepcidin and ferroportin showed a strong positive correlation, suggesting a common regulatory pattern rather than a direct causal relationship.

Available studies assessing the clinical effects of nutritional education on iron status parameters in infants and young children are limited. Most of these studies evaluate hemoglobin concentration alone [29,30] or in combination with iron and ferritin concentrations as the primary indicators for diagnosing anemia [31,32].

In the observational study by Al-Suhiemat et al., a sample of 100 anemic children aged 36–59 months was assessed. The authors examined maternal nutritional knowledge and feeding practices in relation to iron deficiency, while biochemical evaluation was limited to measuring hemoglobin concentrations. The key finding of the study was that children’s hemoglobin levels were significantly associated with maternal nutritional knowledge, particularly regarding the appropriate age for introducing iron-rich complementary foods and the provision of animal-source iron in the diet [29].

In the study by Abu Alhaija et al., 213 infants aged 9–14 months with anemia diagnosed on the basis of low hemoglobin levels were enrolled. The participants were assigned to two groups: an intervention group, in which mothers received a three-month nutritional education program, and a control group that received no educational intervention. The aim of the study was to evaluate the effect of the educational program in terms of changes in mothers’ nutritional knowledge and in infants’ hemoglobin levels. The results showed a statistically significant increase in hemoglobin concentrations in the intervention group, which was accompanied by a reduction in the prevalence of anemia [30].

In the study by Kapur et al., 232 children aged 9–36 months were assigned to four groups: a nutrition education group, a weekly iron supplementation group, a combined nutrition education plus supplementation group, and a placebo control group. The intervention lasted four months and included an eight-week treatment phase followed by eight weeks without treatment. The aim of the study was to compare the effects of nutrition education and iron supplementation—administered separately or in combination—on children’s iron status (hemoglobin, ferritin concentrations). At eight weeks, none of the interventions resulted in a significant change in hemoglobin levels. However, by sixteen weeks, the group receiving nutrition education showed a significant improvement in hemoglobin concentrations and a smaller decline in serum ferritin compared with the control group. It is important to note that, as in our study, the sample did not include only children with anemia. The baseline prevalence of anemia was 57%, meaning that the cohort also included children with normal initial hemoglobin levels [31].

Khoshnevisan et al. evaluated the effect of a three-month nutrition education program in 62 iron-depleted children aged 2 to 6 years. Children were enrolled based on iron, hemoglobin, and ferritin concentrations, as well as transferrin saturation values. The intervention increased ferritin concentrations, while no differences in transferrin saturation were observed before and after the program. The study did not report the effect of the nutritional education intervention on hemoglobin or serum iron concentrations [32].

Available scientific evidence indicates that intensive nutritional education can significantly improve iron status in infants and young children. However, most available studies focus primarily on children already diagnosed with anemia or incorporate iron and/or other nutrient supplementation as part of the intervention. There is a lack of long-term research examining the influence of intervention-based nutritional education on comprehensive iron homeostasis in infants, beyond those diagnosed with anemia.

Preventing the development of anemia in infancy and ensuring its prompt recovery when it occurs is crucial for healthy infant development. According to current knowledge, increasing dietary iron intake is the key nutritional strategy in this context.

Our study demonstrated that nutritional education provided to caregivers can meaningfully increase iron intake in infants, highlighting that implementing preventive measures in such a young population may contribute to a tangible reduction in the incidence of anemia.

It is worth emphasizing that in our study infants were identified primarily on the basis of abnormal iron and ferritin parameters, rather than hemoglobin levels, which remained within the normal range. Therefore, when anemia is suspected in infants and young children, it is advisable to assess the full spectrum of iron status indicators rather than relying solely on hemoglobin concentrations. This is particularly important because iron deficiency may develop before a decline in hemoglobin becomes apparent. Such an approach is consistent with the recommendations of the American Academy of Pediatrics (AAP) and the European Society for Pediatric Gastroenterology, Hepatology and Nutrition (ESPGHAN). According to the recommendations of these societies, the diagnostic evaluation of anemia should include hemoglobin, red blood cell count, hematocrit, white blood cell count, platelet count, and red blood cell indices such as mean corpuscular volume, mean corpuscular hemoglobin, and mean corpuscular hemoglobin concentration. The assessment should also encompass red cell distribution width (RDW), reticulocyte count, and serum ferritin. In situations where an inflammatory state or infection is suspected, measurement of C-reactive protein is additionally recommended. Although such testing should be performed only in the presence of clinical signs, symptoms, or identifiable risk factors—such as peripartum hemorrhage, maternal diabetes, pre-eclampsia, bovine protein allergy, exclusive breastfeeding beyond 6 months, or early introduction of cow’s milk before 1 year of age [33]. However, ESPGHAN does not recommend routine screening for iron deficiency in infants. In contrast, the American Academy of Pediatrics recommends routine hemoglobin-based screening for iron deficiency anemia at 12 months of age [33,34,35].

A limitation of this study was the lack of biochemical assessments during the nutritional intervention (biochemical parameters were performed at the beginning and end of the study). Future research should incorporate repeated measurements to capture temporal changes in relevant parameters and to identify which indicators are most informative for monitoring both short- and long-term responses to anemia treatment in infants. Nevertheless, our study is the first to evaluate the impact of long-term, intensive nutritional education on infant iron status using a broad range of iron-related biochemical parameters together with clinical assessment. Another limitation is the failure to take into account include timing of cord clamping because this information is not routinely provided to the mother in the hospital discharge summary or in the child health record and access to hospital medical records was not available within the study protocol.

## 5. Conclusions

Although there are strong reasons to expect that parental nutritional education could improve iron status in infants, our study—despite extensive and detailed assessment, including hepcidin—did not demonstrate a clear signal of such an effect. Nevertheless, the findings are highly encouraging, as we observed a lower prevalence of iron and ferritin deficiencies among infants whose parents participated in the nutritional education program. This effect appears to be driven by the increased iron intake in the study group.

## Figures and Tables

**Table 1:** Anthropometric data of infants which completed the study at baseline.

Parameter	Study Group (*n* = 52)		Control Group (*n* = 52)		*p*
	Median (Q1–Q3)	Mean ± SD (95% CI)	Median (Q1–Q3)	Mean ± SD (95% CI)	
Infants					
Body weight [g]	5430	5917 ± 1611	6050	5707 ± 1188	0.857
	(4655–7400)	(5292–6543)	(4610–6560)	(5246–6168)	
*Z*-score for body weight at baseline	0.02	0.98 ± 2.50	0.03	−0.03 ± 1.25	0.456
	(−0.97–3.13)	(0.01–1.95)	(−0.49–0.77)	(−0.52–0.45)	
Sex: female	48.08%		51.92%		0.823

Q—quartile; SD—standard deviation; CI—confidence interval. *p*-value for the U Mann–Whitney test or the χ^2^ test.

**Table 2:** Comparison of the probability of occurrence of concentrations of the tested parameters below the reference value limits between the study group and the control group *.

Parameters	Baseline				*p*	Follow-Up				*p*	*p* ^1^	*p* ^2^
	Study Group (*n* = 52)		Control Group (*n* = 52)			Study Group (*n* = 52)		Control Group (*n* = 52)				
	Median (Q1–Q3)	Results <LRL	Median (Q1–Q3)	Results <LRL		Median (Q1–Q3)	Results <LRL	Median (Q1–Q3)	Results <LRL			
RBC (10^12^/L)	3.9 (3.7–4.8)	12	4.2 (3.9–4.5)	7	0.845	4.6 (4.4–4.8)	1	4.5 (4.1–4.6)	5			0.621
HGB (g/dL)	12.0 (11.4–12.6)	0	11.3 (11.0–11.9)	0		11.9 (11.0–12.7)	0	11.4 (10.4–12.1)	0			
HCT (%)	33.4 (31.05–34.8)	12	31.7 (30.0–33.7)	15	0.327	34.9 (33.9–36.7)	10	33.1 (31.1–34.8)	14	0.242	0.595	0.500
MCV (fL)	85.7 (79.1–91.1)	4	75.6 (73.7–76.9)	17		74.6 (73.3–76.9)	24	76.2 (70.9–79.9)	22	0.578		0.209
MCH (pg)	31.1 (28.5–33.1)	5	27.1 (26.5–28.2)	10	0.132	26.0 (25.3–26.7)	11	26.7 (23.4–28.5)	17	0.135	0.087	0.089
MCHC (g/dL)	36.1 (35.5–36.4)	0	35.9 (35.1–36.4)	0		34.4 (33.9–35.0)	8	34.6 (33.3–36.1)	13	0.164		
Iron (µg/dL)	76.3 (51.9–108.4)	23	58.5 (44.5–75.5)	31	0.085	65.0 (52.6–87.0)	13	55.6 (37.1–70.9)	26	**0.008**	**0.031 ^†^**	0.785
Ferritin (ng/mL)	175.5 (71.9–274.8)	7	151.3 (77.5–219.1)	8	0.500	35.1 (27.9–42.4)	8	18.9 (14.9–37.6)	22	**0.003**	0.500	**0.002**
Transferrin (g/L)	2.07 (1.77–2.51)	0	2.33 (2.09–2.65)	0		3.09 (2.82–3.31)	0	3.02 (2.69–3.25)	0			
UIBC (µg/dL)	169.0 (136.5–265.7)	0	222.0 (176.5–261.0)	0		286.0 (259.7–309.7)	1	300.0 (264.5–331.0)	5			
TIBC (µg/dL)	259.0 (226.0–324.2)	0	285.0 (257.5–304.5)	0		356.5 (330.5–379.5)	2	382.0 (293.5–406.5)	5			
Hepcidin (ng/mL)	244.0 (132.6–444.2)		339.3 (239.9–521.0)			729.7 (379.4–902.8)		699.5 (422.7–1076.1)				
Ferroportin (ng/mL)	9.2 (6.4–15.6)		12.7 (9.7–220.9)			22.8 (15.3–28.5)		32.8 (17.5–50.3)				
	Median (Q1–Q3)	**Results** **>URL**	Median (Q1–Q3)	**Results** **>URL**		Median (Q1–Q3)	**Results** **>URL**	Median (Q1–Q3)	**Results** **>URL**			
hsCRP (mg/L)	9.7 (2.8–30.1)	28	15.6 (3.6–35.0)	22	0.837	7.2 (2.2–16.1)	19	17.5 (7.4–40.3)	24	0.213	0.942	0.422

* The analysis was conducted only in cases where at least five observations were below the lower limit of the reference range. Q—quartile; <LRL—the result below the lower limit of the reference range; >URL—the result above the upper limit of the reference range; †—using the opposite assumptions of the alternative hypothesis. RBC—red blood cells (normal value for females and males: 3.7–5.3 × 10^12^/L); HGB—hemoglobin (normal value: 9.5–14.5 g/dL for infants aged 1–3 months and 10.5–13.5 g/dL for infants aged up to 9 months); HCT—hematocrit (normal value: females—32.5–41.0%; males: 27.5–41.0%); MCV—mean cell volume (normal value: 85–100 fL for infants aged 1–3 months and 75–85 fL for infants aged up to 9 months); MCH—mean corpuscular hemoglobin (normal value: females—26.0–28.8 pg; males: 23.0–29.8 pg); MCHC—mean cell hemoglobin concentration (normal value: females—33.8–35.9 g/dL; males: 33.0–35.9 g/dL); iron (normal value: females—75–235 µg/dL; males: 72–203 µg/dL); ferritin (normal value: females 79–501 ng/mL, males 40–755 ng/mL for infants aged 1–3 months and females 25–56 ng/mL, males 25–79 ng/mL for infants aged up to 9 months); transferrin (normal value: females 0.65–1.90 g/L, males 0.70–2.39 g/L for infants aged to 3 months and females 1.35–3.50 g/L, males 1.35–2.60 g/L for infants aged 4–12 months); UIBC—unsaturated iron-binding capacity (normal value: females 40–90 µg/dL, males 83–127 µg/dL for infants aged to 3 months and females 190–263 µg/dL, males 127–238 µg/dL for infants aged 4–12 months); TIBC—total iron binding capacity (normal value: females 165–275 µg/dL, males 155–330 µg/dL for infants aged to 3 months and females 250–455 µg/dL, males 150–380 µg/dL for infants aged 4–12 months); hepcidin and ferroportin—no standardized, widely accepted normal values for infants (data mainly from research studies); hsCRP—high-sensitivity C-reactive protein (normal value: >10 mg/L). Normal values for: HGB and MCV according to [24], for iron, transferrin, UIBC and TIBC according [25]. For other parameters, normal values were adopted according to the manufacturer’s guidelines for the haematology analyzer. *p—p* value for the comparison of proportions between the study group and the control group. *p*^1^—*p* value for the comparison of proportions between Baseline and Follow-up in the study group. *p*^2^—*p* value for the comparison of proportions between Baseline and Follow-up in the control group. Statistically significant differences are highlighted in bold.

**Table 3:** Correlation between iron and other parameters in infants with iron concentrations below the reference range.

		Iron
Ferritin (ng/mL)	Spearman’s rho	0.003
	*p*-value	0.996
Transferrin (g/L)	Spearman’s rho	−0.003
	*p*-value	0.991
UIBC (µg/dL)	Spearman’s rho	−0.009
	*p*-value	0.974
TIBC (µg/dL)	Spearman’s rho	0.175
	*p*-value	0.516
Hepcidin (ng/mL)	Spearman’s rho	0.135
	*p*-value	0.617
Ferroportin (ng/mL)	Spearman’s rho	0.094
	*p*-value	0.730
hsCRP (mg/L)	Spearman’s rho	−0.724 *
	*p*-value	**0.002**
RBC (10^12^/L)	Spearman’s rho	0.026
	*p*-value	0.926
HGB (g/dL)	Spearman’s rho	0.175
	*p*-value	0.516
HCT (%)	Spearman’s rho	0.256
	*p*-value	0.338
MCV (fL)	Spearman’s rho	0.271
	*p*-value	0.310
MCH (pg)	Spearman’s rho	0.194
	*p*-value	0.471
MCHC (g/dL)	Spearman’s rho	−0.092
	*p*-value	0.736

Note. * *p* < 0.01 Statistically significant differences are highlighted in bold.

## Data Availability

The datasets generated and analyzed during the current study are available from the corresponding author upon reasonable request. The data are not publicly available due to ethical restrictions, in particular those related to the protection of personal data and the privacy of the study participants.
